# The Importance of GLWamide Neuropeptides in Cnidarian Development and Physiology

**DOI:** 10.4061/2011/424501

**Published:** 2011-10-20

**Authors:** Toshio Takahashi, Masayuki Hatta

**Affiliations:** ^1^Suntory Foundation for Life Sciences, Bioorganic Research Institute, 1-1-1 Wakayamadai, Shimamoto, Mishima, Osaka 618-8503, Japan; ^2^Department of Biology, Ochanomizu University, Bunkyo-ku, Tokyo 112-8610, Japan

## Abstract

The peptide-signaling molecules (<50 amino acid residues) occur in a wide variety of invertebrate and vertebrate organisms, playing pivotal roles in physiological, endocrine, and developmental processes. While some of these peptides display similar structures in mammals and invertebrates, others differ with respect to their structure and function in a species-specific manner. Such a conservation of basic structure and function implies that many peptide-signaling molecules arose very early in the evolutionary history of some taxa, while species-specific characteristics led us to suggest that they also acquire the ability to evolve in response to specific environmental conditions. In this paper, we describe GLWamide-family peptides that function as signaling molecules in the process of muscle contraction, metamorphosis, and settlement in cnidarians. The peptides are produced by neurons and are therefore referred to as neuropeptides. We discuss the importance of the neuropeptides in both developmental and physiological processes in a subset of hydrozoans, as well as the potential use as a seed compound in drug development and aspects related to the protection of corals.

## 1. Introduction

Peptide-signaling molecules (<50 amino acid residues) have been found in a wide variety of organisms and many are known to play important roles in regulating physiological processes in both vertebrates and invertebrates. For example, peptide-signaling molecules act as neurohormones in the endocrine system and as neurotransmitters or neuromodulators in the nervous system. In addition, individual neuropeptides have been found to be multifunctional and may be involved in the immune response, developmental processes, and physiological processes within a single organism. The action of these peptides has been found to be spatio-temporally regulated, which ensures that the timing and pattern of development proceed correctly and that viability is maintained. 

**Figure 1:**
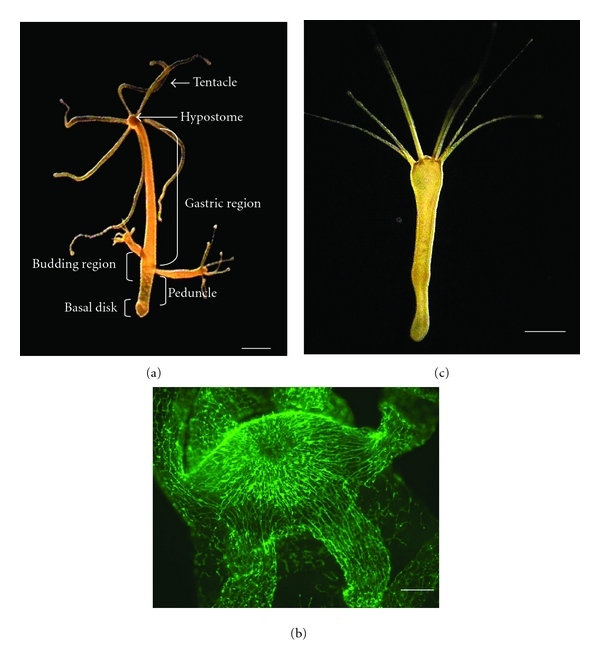
Types of hydra. (a) Normal *Hydra*. (b) Head of *Hydra*. Nerve cells are visualized by indirect immunofluorescence using an anti-RFamide antibody. (c) Epithelial *Hydra*. Scale bars represent 2 mm (a and c) and 100 *μ*m (b), respectively.

 Among metazoan organisms, the freshwater cnidarian *Hydra *(Figure 1(a)) has one of the most primitive nervous systems. The nervous system of the *Hydra* is generally regarded as a diffuse, net-like structure that extends throughout the animal (Figure 1(b)). This “nerve net” is composed of two morphologically distinct cell types, ganglion cells, and sensory cells [1]. To date, a variety of neuropeptides have been identified in Cnidaria. For example, the GLWamide-family peptides that have been isolated from the sea anemone, *Anthopleura elegantissima *[2], and *Hydra magnipapillata *[3] have been shown to induce the metamorphosis of* Hydractinia serrata *planula larvae into polyps. In *Hydra,* GLWamides induce detachment of the bud from a parental polyp [3]. Budding is the form of asexual reproduction in this animal. The neuropeptide, Hym-355 that Hym are tentatively named from *Hydra magnipapillata*, enhances neuronal differentiation by inducing multipotent interstitial stem cells to enter the neuron differentiation pathway [4]. A myoactive neuropeptide, Hym-176, specifically and reversibly induces contraction of the ectodermal muscles of the body column, in particular in the peduncle region of *Hydra* [5]. In addition, one of the Hydra-RFamides, Hydra-RFamide III, has a dose-dependent effect on pumping of the peduncle, which is considered to be the equivalent of the heart of higher organisms [6]. More recently, two members of a novel neuropeptide family (FRamides) that form part of the same precursor were shown to have opposite myoactive functions in epithelial *Hydra* (Figure 1(c)), which are *Hydra* that have no nerve cells [7].

GLWamide-family peptides have characteristic structural features in their N- and C-terminal regions. For example, the peptides all share a GLWamide motif at their C-termini, and seven of the GLWamide peptides isolated from *Hydra* were found to have a proline residue at the second position (X-Pro) or at the second and third positions (X-Pro-Pro) of their N-terminal regions (Table 1). Metamorphosin A (MMA), isolated from *Anthopleura*, has a pyroglutamyl residue at the N-terminus. Interestingly, both of these N-terminal structures confer resistance to aminopeptidase digestion [8].

 Recently, one of the *Hydra*-derived GLWamide-family neuropeptides, Hym-248, was demonstrated to induce both settlement and metamorphosis in cultured planulae of nine Pacific *Acropora* species and mixed *Acropora* coral slicks with remarkable consistency [9, 10]. Furthermore, planulae of the Caribbean coral *Acropora palmata* were induced to settle by the exogenous application of Hym-248 [11]. These observations suggest that the underlying neurotransmission signal, which is induced by external cues, is conserved and can be exogenously manipulated beyond hydrozoans. 

 In this paper, we describe GLWamide-family peptides that function as signaling molecules in muscle contraction, metamorphosis, and settlement in cnidarians including corals and polyps. We also discuss the importance of the neuropeptides in the development and physiology of a subset of hydrozoans and also their potential role as seed compounds in drug discovery and aspects related to the protection of corals.

## 2. Induction of Metamorphosis in Planula Larvae of the Marine Hydrozoan *Hydractinia*

**Figure 2:**
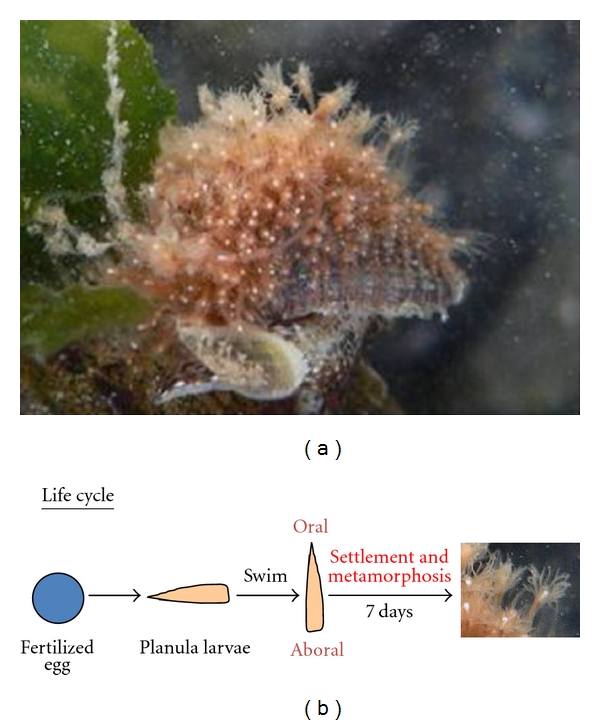
*Hydractinia*. (a) *Hydractinia epiconcha* on a hermit crab. (b) Life cycle of *Hydractinia*.

Species of the genus *Hydractinia* are colonial and usually live on snail shells inhabited by hermit crabs (Figure 2(a)). In their life cycle, there is only a planula larval stage with no medusa stage (Figure 2(b)). Upon settling, the planula larvae undergo metamorphosis and develop into polyps after approximately one week. Leitz et al. [2] isolated the neuropeptide MMA from the sea anemone *Anthopleura elegantissima* and showed that MMA induces the metamorphosis of planula larvae of the marine hydrozoan *Hydractinia echinata* into polyps (Table 1). Their findings demonstrated that, in addition to playing roles as neurotransmitters and neuromodulators, some cnidarian neuropeptides function as neurohormones and control developmental processes. We independently isolated seven peptides belonging to the GLWamide family from *Hydra magnipapillata* that shared a GLWamide motif in their C-terminal region (Table 1) [3, 12]. 

**Figure 3:**
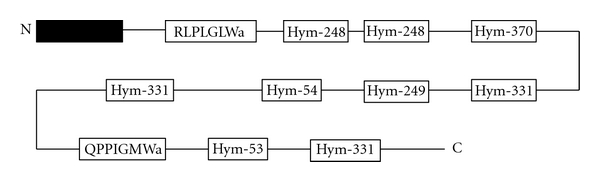
Schematic representation of the preprohormone containing a signal sequence (black box) and two putative neuropeptide sequences (RLPLGLWamide and QPPIGMWamide) and unprocessed GLWamide peptides (Hym-53, 54, 248, 249, 331, and 370) in *Hydra*.

All neuropeptides are produced and secreted by highly regulated secretion pathways. In general, the precursors of neuropeptides are incorporated into the endoplasmic reticulum as a preprohormone where they are converted into prohormones via the removal of a signal peptide region. These prohormones are then transported to the Golgi apparatus where they undergo posttranslational modification such as endoproteolysis and C-terminal amidation before assuming their final active peptide forms [13]. Leviev et al. cloned the *Hydra* preprohormone gene that encodes all of the GLWamide peptides that we identified previously (Figure 3) [14]. 

 All of the GLWamide peptides produced by *Hydra* have the ability to induce the metamorphosis of *Hydractinia serrata* planula larvae into polyps [3, 12]. An N-terminal deletion series revealed that a common GLWamide sequence is necessary for the induction of metamorphosis in *Hydractinia*. However, the precise mechanisms involving the GLWamide peptides in the induction of metamorphosis are not yet clearly understood. Interestingly, larvae can be induced to undergo metamorphosis in response to a chemical signal secreted by environmental bacteria [2]. This chemical signal is most likely received by the sensory neurons of the planula larvae, which then release GLWamide peptides that act on the surrounding epithelial cells and result in a change in the phenotype. However, since hydras develop directly from embryos into adult polyps and have no intermediate larval stage, the precise function of the GLWamide peptides in early embryogenesis in *Hydra* is thus still an open question.

## 3. Myoactivity in *Hydra* and the Sea Anemone *Anthopleura*

**Figure 4:**
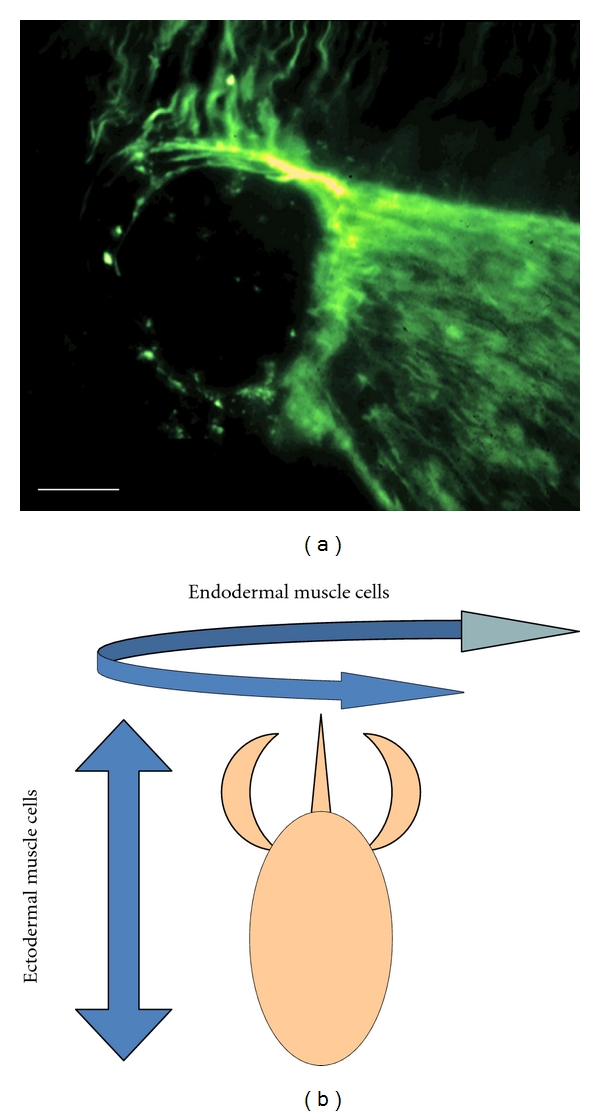
Muscle process in hydra. (a) Sphincter muscle process in bud was stained using antimyosin heavy chain. Scale bar represents 0.5 mm. (b) Muscle processes of ectodermal epithelial cells run longitudinally and those of endodermal epithelial cells run circumferentially.

Given the absence of a planula larval stage in the life cycle of *Hydra*, we searched for another function of GLWamide peptides in this genus. Interestingly, we found that all GLWamide peptides induce detachment of the bud from the parental polyp [3]. Briefly, the process of budding is associated with the development of a circular or sphincter muscle in the basal disk late in bud development (R. Campbell, *pers. comm.*) (Figure 4(a)). Contraction of this sphincter muscle results in the constriction of the basal disk of the bud, thereby “pinching off” the bud from the parent. Tests of myoactivity typically employ epithelial *Hydra* (Figure 1(c)), which are hydras with no nerve cells or any of the other cells derived from the interstitial stem cell lineage, except gland cells [15, 16]. Since epithelial *Hydra* are primarily composed of epithelial muscle cells, the epithelial layer of the *Hydra* “body” is a homogenous, *in vivo* muscle preparation that is particularly well suited to examinations of the direct effect of a peptide on muscle cells. A similar effect, that is, induction of bud detachment, was also observed in normal *Hydra* treated with the peptides.

 While elucidating the function of the GLWamide-family peptides, we found that Hym-248 had an unexpected effect on the *Hydra* body. Specifically, Hym-248 not only induced bud detachment when applied to epithelial *Hydra*, but also elongated the body column [12]. In *Hydra*, muscle processes extending from ectodermal and endodermal epithelial cells run perpendicular to each other, with the ectodermal processes oriented along the body column axis and endodermal processes oriented circumferentially (Figure 4(b)). Contraction of the ectodermal muscles results in a shortening of the body column, while contraction of the endodermal muscles, such as that observed in response to Hym-248, results in elongation of the body column. Since Hym-248 has two target muscles (the sphincter and endodermal muscles), it is possible that Hym-248 has two types of receptors: one that is common to all GLWamide-family peptides and another is specific to Hym-248. In higher organisms, most neuropeptides act as ligands for G-protein-coupled receptors (GPCRs) on target cells. Signaling via GPCR is a major route of cellular communication via the plasma membrane. Identification of both the common receptor of GLWamide-family peptides and the Hym-248-specific receptor is currently in progress.

**Figure 5:**
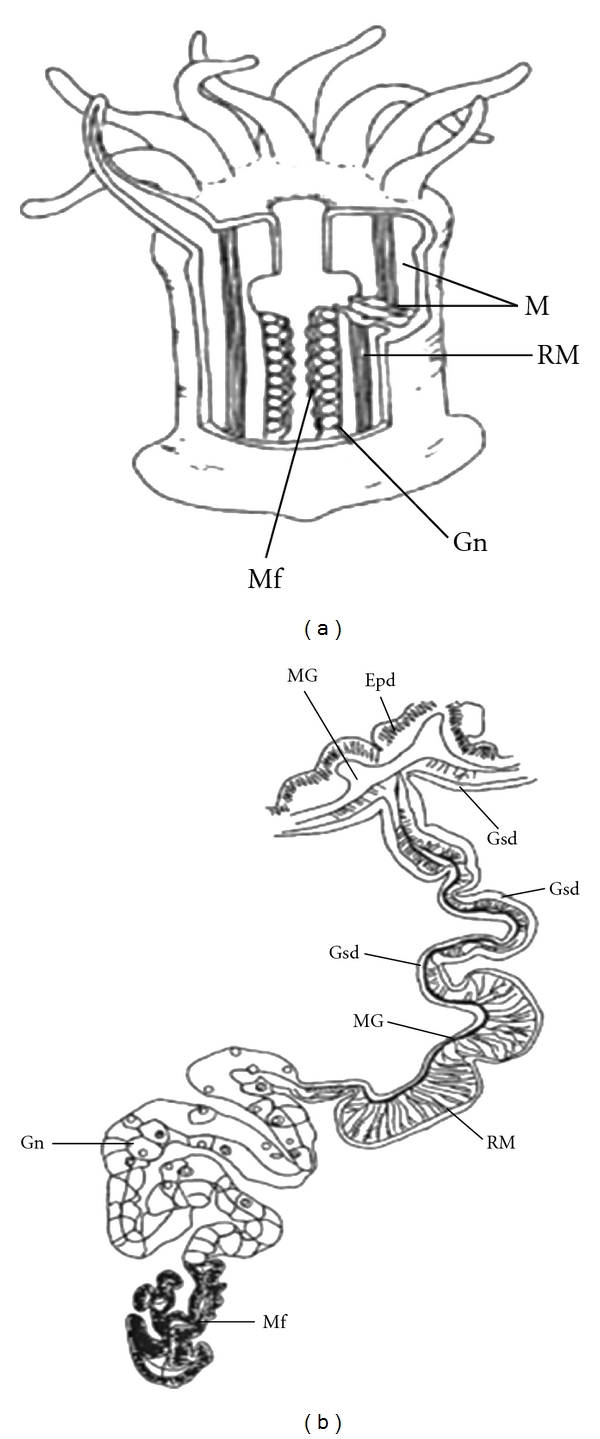
Schematic diagram of *A. fuscoviridis* and its retractor muscle. (a) Longitudinal section of the sea anemone showing the paired mesenteries that partition the gastric cavity. The free edge of the mesentery below the pharynx contains the gonads and filaments. The longitudinal retractor muscles are located on one side of the mesentery. (b) Detailed illustration of a cross-section through a mesentery. A mesentery is composed of two gastrodermal layers, one containing the longitudinal retractor muscles. The mesoglea ramifies within the retractor muscles. M, paired mesenteries; RM, retractor muscle; Epd, epidermis (ectoderm); Gsd, gastrodermis (endoderm); MG, mesoglea; Gn, gonad; Mf, mesenterial filament. Comparative Biochemistry and Physiology Part B (2003) 135, 309-324 [12].

The isolation of MMA from the sea anemone *Anthopleura elegantissima* implies that GLWamide-family peptides are biologically active in this organism. We found that all GLWamide-family peptides induced contraction of the retractor muscle (RM) in *Anthopleura fuscoviridis *[12]. The gastric cavity of *A. fuscoviridis* is partitioned by pairs of mesenteries, which are longitudinal extensions of the body wall that are covered by gastrodermal epithelium (Figure 5). The free apical edges of the mesenteries contain the gonads and the filaments, and the RMs are located proximal to these structures (Figure 5(b)), which extend longitudinally from the oral disk to the basal disk on one side of each mesentery (Figure 5). These muscles are the strongest and most well developed in *A. fuscoviridis*. Immunohistochemical staining with an antibody specific for the GLWamide motif revealed intensely stained nerve cells in the RMs of the sea anemone as well as the nervous system of *Hydra *[12]. These observations corroborated those of Leitz and coworkers who reported similar findings in *Hydra* and *Hydractinia* [17, 18]. Taken together, these findings imply that GLWamide-family peptides act as neurotransmitters or neuromodulators at neuro-muscular junctions in Cnidaria.

**Figure 6:**
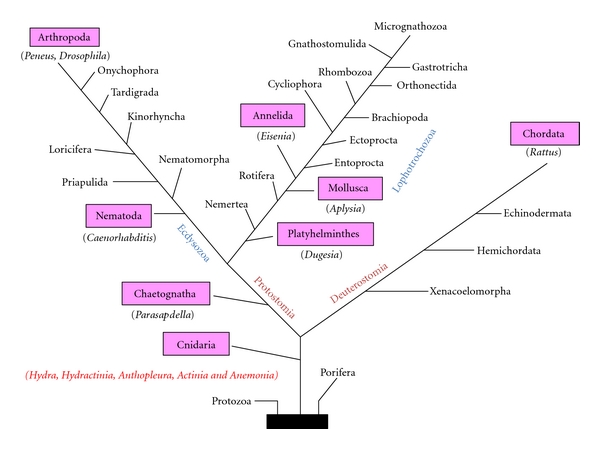
Phyla with anti-GLWamide antibody-positive neurons. Immunoreactive phyla are colored in pink.

 To determine whether members of the GLWamide family occur in other phyla, we screened a variety of organisms for the GLWamide motif using an anti-GLWamide antibody (Figure 6). In almost all cases, neurons were observed to be immunoreactive (Fujisawa and Koizumi, unpublished observations). While this does not necessarily mean that GLWamide-related peptides are present in these species, it does suggest that, like the RFamide superfamily [19], GLWamide-related peptides are widely distributed throughout the animal kingdom. However, at least in C. *elegans*, a gene encoding three putative GLWamide peptides exists.

## 4. Settlement and Metamorphosis Induction in the Coral *Acropora*

Coral reef ecosystems are important components of shallow tropical sea environments. Nonetheless, despite the implementation of a variety of preservation and management initiatives, the effects of direct and indirect anthropogenic activities are destroying coral reefs at an alarming rate. The most conspicuous example of this damage is coral bleaching, which occurs as when the intracellular symbiotic algae (zooxanthellae) of the choral die [20]. Over the last two decades, coral bleaching has increased in frequency, intensity, and spatial extent [21]. 

**Figure 7:**
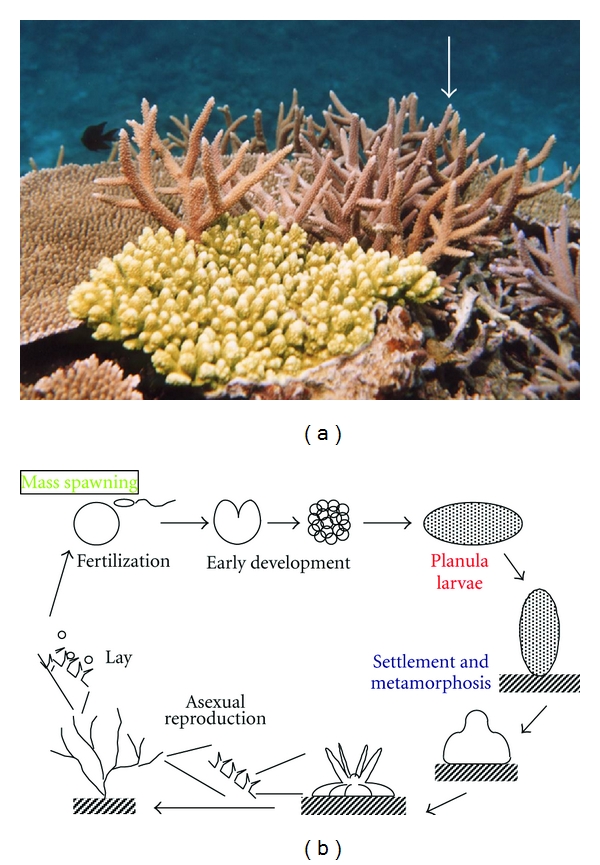
Appearance and life cycle of *Acropora*. (a) *Acropora* spp. (arrow), (b) life cycle of* Acropora*.

 In Indo-Pacific reefs, the coral genus *Acropora* (Figure 7(a)) consists of major species and coral communities are maintained in large part through recruitment of their larvae. Sexual reproduction in many acroporids occurs via “mass spawning” events, which are characterized by the synchronous release of millions of gametes into the water column by many colonies belonging to multiple species [22, 23]. The high densities of floating gametes form patches referred to as “slicks” on the ocean surface. The resultant larvae are then dispersed by currents where after they settle on substrates and begin the sedentary part of their life cycle (Figure 7(b)). Despite the large number of larvae produced by these mass spawning events, very few survive the drifting period and only a small proportion are recruited into reef communities. If some of the larvae that would otherwise be lost prior to settlement could be collected and cultivated under controlled conditions, they could be used as donors for reef transplantation without damaging existing coral communities.

 However, the difficulty associated with such an approach is the lack of a method for controlling the settlement and metamorphosis of *Acropora*. We recently found that a member of the GLWamide family of peptides, Hym-248, is capable of inducing metamorphosis of acroporid larvae into polyps at high rates (approximately 100%) [10] and that *Acropora* planulae responded to the peptide in a concentration-dependent manner [10]. Planulae of the Caribbean coral *Acropora palmata* were also induced to settle after the exogenous application of Hym-248, with rates of attachment and metamorphosis of planula larvae of six days after fertilization reaching 40–80% and 100%, respectively [11]. Interestingly, however, Hym-248 was not capable of inducing metamorphosis in other coral genera [10, 11]. It therefore appears that, in corals, the specificity of ligand recognition by receptors is dependent on the extent to which peptide(s) of particular structures are recognized. However, in *Hydractinia*, the specificity is less stringent and receptors are able to recognize any peptides belonging to the GLWamide family. Since Hym-248 is a surrogate ligand in *Acropora*, natural ligand(s) that are similar in structure to Hym-248 should be identified.

 This is the useful success in inducing metamorphosis of coral larvae in *Acropora*. Indeed, Hatta and Iwao [9] have started assessing the potential application of the peptide to “coral seeding,” by collecting coral larvae after mass spawning events and producing primary polyps or infant colonies for the purpose of transplantation and the subsequent restoration of coral reefs.

## 5. Conclusions

The role of peptides as important signal molecules in the development and physiology of primitive metazoans, such as *Hydra*, has long been recognized. In this paper, we described those GLWamide-family peptides that have been identified and isolated from sea anemones and hydra over the course of the *Hydra* Peptide Project that was initiated by us as a systematic peptide screening project to identify novel peptide molecules involved in regulation of development and physiological processes in *Hydra* [3]. These peptides have been shown to induce metamorphosis in both *Hydractinia* and *Acropora* planula larvae, and they also exhibit myoactivity in both hydra and sea anemone. While these peptides appear to be multifunctional, it is not apparent from the effects of the peptides in the same species. In addition, specific receptors of GLWamide-family peptides, such as Hym-248, have not yet been identified. Recently, the ability with which genes encoding peptide receptors, particularly GPCRs, has been greatly enhanced by the Hydra EST Project [24] and sequencing of the *Hydra* genome [25]. By mining these databases, it is hoped that specific GPCR receptors will be found in the near future.

 It is well known that GLWamide-related peptides are present in higher metazoans, and GLWamide-like immunoreactivity has been observed in the cell bodies of neurons and in thin varicose fibers in some regions of rat brain [26]. These results strongly suggest that the rat nervous system contains as yet unidentified GLWamide-like peptides. Regarding the likely function of these peptides, the distribution patterns of GLWamide-positive fibers are similar to those of substance P, which is considered to be an important neuropeptide in the processing of nociceptive information [26]. Taken together, these findings imply that rat GLWamide-like peptides are involved in sensory mechanisms, possibly those related to nociception. The search for authentic mammalian GLWamide-like peptides is currently in progress.

 Finally, we also discussed potential applications of the GLWamide-family peptides. For example, GLWamide-family peptides appear to occur in a variety of animal taxa, ranging from mammals to cnidarians. One such application is the use of Hym-248 to produce “coral seedlings” which can then be transplanted and used for reef restoration. Other potential applications include the development of novel drugs that exploit the GLWamide motif to treat nociceptive pain in the mammalian brain.

## Figures and Tables

**Table 1:** Structure and function of GLWamide-family peptides isolated from *Hydra* and *Anthopleura*.

Name	Structure	Function
Hym-53	NPYPGLWamide	Myoactivity and Metamorphosis
Hym-54	GPMTGLWamide	Myoactivity and Metamorphosis
Hym-248	EPLPIGLWamide	Myoactivity, Metamorphosis, and Settlement
Hym-249	KPIPGLWamide	Myoactivity and Metamorphosis
Hym-331	GPPPGLWamide	Myoactivity and Metamorphosis
Hym-338	GPP^h^PGLWamide	Myoactivity and Metamorphosis
Hym-370	KPNAYKGKLPIGLWamide	Myoactivity and Metamorphosis
MMA	pQQPGLWamide	Myoactivity and Metamorphosis

^h^P: hydroxyproline. pQ: pyroglutamate.

## References

[1] David CN (1973). A quantitative method for maceration of *hydra* tissue. *Wilhelm Roux Archiv für Entwicklungsmechanik der Organismen*.

[2] Leitz T, Morand K, Mann M (1994). Metamorphosin A: a novel peptide controlling development of the lower metazoan *Hydractinia echinata*. *Developmental Biology*.

[3] Takahashi T, Muneoka Y, Lohmann J (1997). Systematic isolation of peptide signal molecules regulating development in *hydra*: LWamide and PW families. *Proceedings of the National Academy of Sciences of the United States of America*.

[4] Takahashi T, Koizumi O, Ariura Y (2000). A novel neuropeptide, Hym-355, positively regulates neuron differentiation in *Hydra*. *Development*.

[5] Yum S, Takahashi T, Koizumi O (1998). A novel neuropeptide, Hym-176, induces contraction of the ectodermal muscle in *Hydra*. *Biochemical and Biophysical Research Communications*.

[6] Shimizu H, Fujisawa T (2003). Peduncle of *Hydra* and the heart of higher organisms share a common ancestral origin. *Genesis*.

[7] Hayakawa E, Takahashi T, Nishimiya-Fujisawa C, Fujisawa T (2007). A novel neuropeptide (FRamide) family identified by a peptidomic approach in *Hydra* magnipapillata. *FEBS Journal*.

[8] Carstensen K, Rinehart KL, McFarlane ID, Grimmelikhuijzen CJP (1992). Isolation of Leu-Pro-Pro-Gly-Pro-Leu-Pro-Arg-Pro-NH2 (Antho-RPamide), an N-termally protected, biologically active neuropeptide from sea anemones. *Peptides*.

[9] Hatta M, Iwao K, Sexena N (2002). Metamorphosis induction and its possible application to coral seedlings production. *Recent Advances in Marine Science and Technology*.

[10] Iwao K, Fujisawa T, Hatta M (2002). A cnidarian neuropeptide of the GLWamide family induces metamorphosis of reef-building corals in the genus *Acropora*. *Coral Reefs*.

[11] Erwin PM, Szmant AM (2010). Settlement induction of Acropora palmata planulae by a GLW-amide neuropeptide. *Coral Reefs*.

[12] Takahashi T, Kobayakawa Y, Muneoka Y (2003). Identification of a new member of the GLWamide peptide family: physiological activity and cellular localization in cnidarian polyps. *Comparative Biochemistry and Physiology B*.

[13] von Eggelkraut-Gottanka R, Beck-Sickinger AG (2004). Biosynthesis of peptide hormones derived from precursor sequences. *Current Medicinal Chemistry*.

[14] Leviev I, Williamson M, Grimmelikhuijzen CJP (1997). Molecular cloning of a preprohormone from *Hydra* magnipapillata containing multiple copies of *Hydra*-LWamide (Leu-Trp-NH_2_) neuropeptides: evidence for processing at Ser and Asn residues. *Journal of Neurochemistry*.

[15] Marcum BA, Campbell RD (1978). Development of *hydra* lacking nerve and interstitial cells. *Journal of Cell Science*.

[16] Campbell RD (1976). Elimination of *Hydra* interstitial and nerve cells by means of colchicine. *Journal of Cell Science*.

[17] Leitz T, Lay M (1995). Metamorphosin A is a neuropeptide. *Roux’s Archives of Developmental Biology*.

[18] Schmich J, Rudolf R, Trepel S, Leitz T (1998). Immunohistochemical studies of GLWamides in Cnidaria. *Cell and Tissue Research*.

[19] Espinoza E, Carrigan M, Thomas SG, Shaw G, Edison AS (2001). A statistical view of FMRFamide neuropeptide diversity. *Molecular Neurobiology*.

[20] Hoegh-Guldberg O (1999). Climate change, coral bleaching and the future of the world’s coral reefs. *Marine and Freshwater Research*.

[21] Huppert A, Stone L (1998). Chaos in the Pacific’s Coral reef bleaching cycle. *American Naturalist*.

[22] Babcock RC, Bull GD, Harrison PL (1986). Synchronous spawnings of 105 scleractinian coral species on the Great Barrier Reef. *Marine Biology*.

[23] Hayashibara T, Shimoike K, Kimura T (1993). Patterns of coral spawning at Akajima Island, Okinawa, Japan. *Marine Ecology Progress Series*.

[24] Fujisawa T, Hayakawa E, Takahashi T, Shimohigashi Y (2004). Systematic identification of peptide signaling molecules by combining *Hydra* peptide and EST projects. *Peptide Chemistry*.

[25] Chapman JA, Kirkness EF, Simakov O (2010). The dynamic genome of *Hydra*. *Nature*.

[26] Hamaguchi-Hamada K, Fujisawa Y, Koizumi O, Muneoka Y, Okado N, Hamada S (2009). Immunohistochemical evidence for the existence of novel mammalian neuropeptides related to the *Hydra* GLW-amide neuropeptide family. *Cell and Tissue Research*.

